# Dedifferentiated fat cells: current applications and future directions in regenerative medicine

**DOI:** 10.1186/s13287-023-03399-0

**Published:** 2023-08-21

**Authors:** Zhuokai Liang, Yufei He, Haojing Tang, Jian Li, Junrong Cai, Yunjun Liao

**Affiliations:** Department of Plastic and Reconstructive Surgery, Nanfang Hospital, Southern Medical University, Guangzhou, 510515 China

**Keywords:** Dedifferentiated fat cells, Regenerative medicine, Cell therapy, Differentiation, Secretion, Redifferentiation

## Abstract

Stem cell therapy is the most promising treatment option for regenerative medicine. Therapeutic effect of different stem cells has been verified in various disease model. Dedifferentiated fat (DFAT) cells, derived from mature adipocytes, are induced pluripotent stem cells. Compared with ASCs and other stem cells, the DFAT cells have unique advantageous characteristics in their abundant sources, high homogeneity, easily harvest and low immunogenicity. The DFAT cells have shown great potential in tissue engineering and regenerative medicine for the treatment of clinical problems such as cardiac and kidney diseases, autoimmune disease, soft and hard tissue defect. In this review, we summarize the current understanding of DFAT cell properties and focus on the relevant practical applications of DFAT cells in cell therapy in recent years.

## Introduction

In recent years, stem cell-based therapeutics have gained great attention due to their wide applications in various degenerative diseases, injuries, and other health-related conditions. Stem cells from different origins, including bone marrow stem cells (BMSCs), cord blood- or adipose tissue-derived mesenchymal stem cells (ASCs), embryonic stem cells (ESCs), and more recently, induced pluripotent stem cells (iPSCs), have been widely reported in many preclinical and clinical studies with some promising results. [1–3]. BMSCs and ASCs are the most popular source of stem cells in current stem cell therapy and tissue engineering research [4]. Yet, BMSCs and ASCs still have their own limitations. For example, the process of bone marrow aspiration is invasive and yields a limited quantity of cells, which is far from adequate for therapy [1–4], and BMSCs are prone to aging after in vitro passage [5]. In the meantime, ASCs can be harvested in a minimally invasive manner. However, ASCs are a heterogeneous cell population [6]. Using a heterogeneous cell population that contains cells at various differentiation stages leads to variations in research and transplantation outcomes.

Dedifferentiated fat (DFAT) cells are derived from the most abundant mature adipocytes in the human body by in vitro “ceiling culture” and have characteristics similar to those of adult stem cells [7–9]. Existing studies have shown that DFAT cells have the characteristics of abundant sources, high homogeneity, strong proliferation ability, adipogenic and osteogenic abilities, low requirements for donor age, and low immunogenicity [8–11]. Compared with adult stem cells such as BMSCs and ASCs, it can better meet the needs of stem cell transplantation therapy and industrial production. This article summarizes the research progress of DFAT cells from the aspects of the source, biological characteristics, and related application research of cell therapy.

## Mature adipocyte

**Fig. 1:**
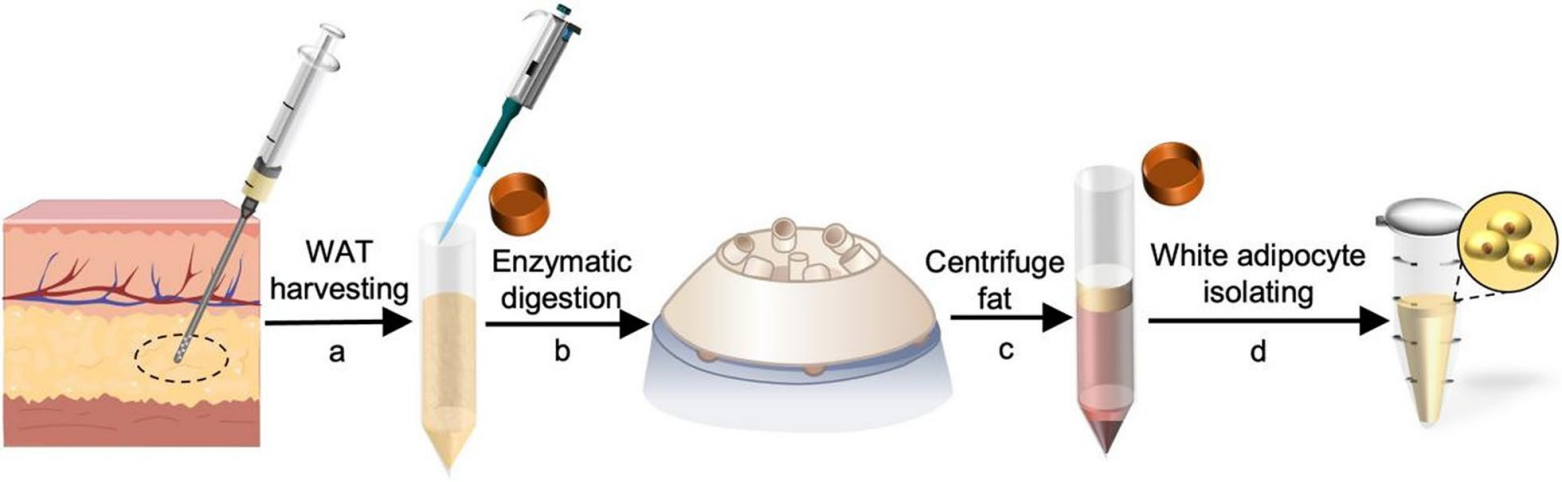
Subcutaneous white adipose tissue (WAT) harvesting and white adipocyte isolation. **a** Harvesting WAT from the clinic; **b** Enzymatic digestion; **c** Centrifuge fat; **d** Isolating white adipocyte. In Fig. 1, all the images except for "the skin structure" sourced from https://biorender.com/ were self-described

The source of DFAT cells is mainly mature adipocytes (MAs) distributed in subcutaneous adipose tissue throughout the body. Subcutaneous white adipose tissue (WAT) could be harvested easily through liposuction and processed into DFAT cells (Fig. 1) [12]. MAs constitute approximately one-third of the cells within WAT that form a storage unit for energy in the form of triglycerides (synthesized from fatty acids) that are packaged into lipid droplets, and that secrete adipokines to regulate animal physiology [13–16]. Adipocytes are plastic and, in response to changes in metabolism, they may change their size, cellular function and even their identity [17–19]. Notably, numerous studies in recent years have observed that adipocyte dedifferentiation and transdifferentiation occur under certain pathophysiological conditions [20–22]. For instance, it has been observed that MAs can be triggered to undergo a thermogenic transformation, resulting in the emergence of beige adipocytes, through exposure to prolonged cold or stimulation of beta-adrenergic receptors, a process commonly referred to as "browning" [23]. Conversely, under conditions of ischemic hypoxia or mechanical stress, MAs are capable of undergoing dedifferentiation into preadipocyte-like cells. [24–26]. Under physiological conditions, adipocytes in mammary glands can undergo lipid loss and revert to preadipocyte-like cells during late pregnancy and lactation [27]. These findings demonstrate that MAs have high plasticity and could undergo dedifferentiation under certain stimuli and induction.

## Induction and identification of DFAT cells

### Induction of DFAT cells

The “ceiling culture” of MAs for the induction of DFAT cells was first proposed by Sugihara in 1986 [28]. Unlike most cell types, MAs are floating cells during in vitro culturing, and the adipocytes would adhere to the upper wall of the culture flask. During ceiling culturing, it was found that adipocytes gradually shed lipid droplets and become fibroblast-like DFAT cells [11].

**Fig. 2:**
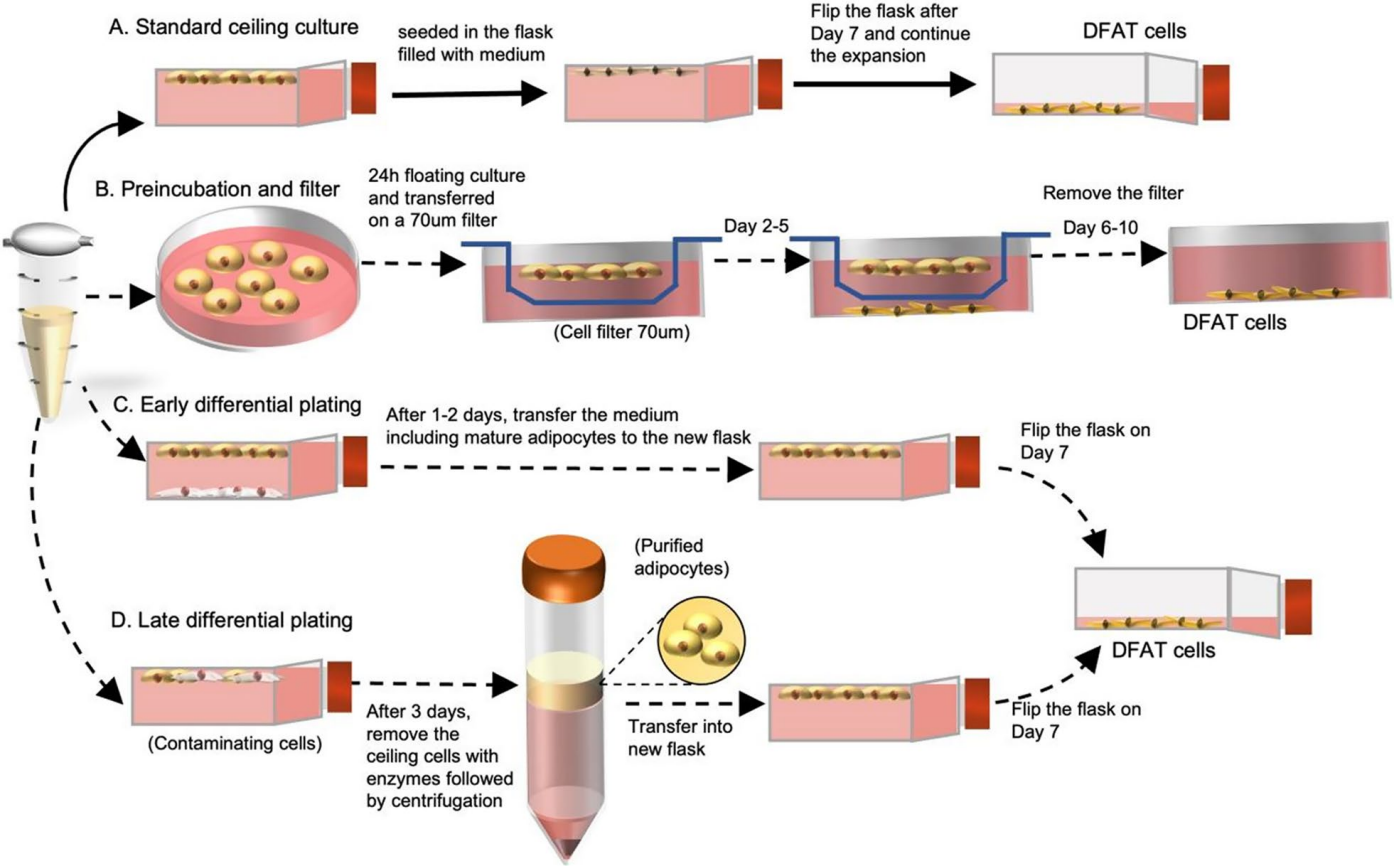
The method of classic “ceiling culture” and its offshoots. **A** system “ceiling culture”: mature adipocytes (MAs) are seeded in a cell culture flask that is completely filled with medium. After 7 days of cell attachment, the flask was inverted and fresh medium was added to barely cover the bottom of the flask to allow cells to continue to grow; **B** Preincubation and filter: After a 24-h floating culture period, MAs were transferred to a new petri dish equipped with a 70-μm cell filter. Subsequently, the DFAT cells gradually migrated through the filter and adhered to the bottom of the dish, where they grew and expanded; **C** Early differential plating: After 1–2 d of ceiling culture, mature adipocytes will be floating in the medium but non–lipid containing cells will attach to the bottom surface. Then floating mature adipocytes in the medium are transferred to the new flask leaving the attached contaminating cells behind; **D** Late differential plating: Mature adipocytes adhere to the top surface after 3–4 d of ceiling culture. Then the medium is removed and the trypsin of the adherent mature adipocytes are digested and centrifuged, to eliminate the contaminating cells culturing along with mature adipocytes by the buoyant nature of adipocytes. The images presented in Fig. 2 were generated and illustrated by the author

Different culture systems to induce DFAT cells are shown in Fig. 2. In the standard celling protocol (Fig. 2A), adipose tissue is minced into small pieces and then dissociated in a 0.1% (w/v) collagenase type I solution at 37 °C for 1 h with gentle agitation. The cell suspension is filtered with a nylon mesh to allow cells to pass through and exclude unwanted tissue remnants. The cell filtrate is centrifuged at 135 g for 3 min to separate cells of the adipose tissue into an upper layer containing MAs and a bottom layer containing cells of the stromal vascular fraction (SVF). MAs remaining afloat on the top layer are collected and seeded in a cell culture flask that is completely filled with Dulbecco’s modified Eagle’s medium supplemented with 20% (v/v) fetal bovine serum (FBS). The fat cells are then incubated at 37 °C with 5% CO2. After the cells were firmly attached and fibroblast-like cells were observed (about 7d), fresh medium is added to barely cover the bottom of the flask to allow cells to continue their expansion [8, 11, 29–31]. According to a previous study [11], it was found that 5 × 10^4^ mature adipocytes can be expanded to produce 3 × 10^6^ DFAT cells. Considering clinical and feasibility aspects, it is possible to isolate sufficient DFAT cells from less than 0.1 g (or 100 mg) of subcutaneous adipose tissue in humans, which yields 4–6 × 10^6^ mature adipocytes. The doubling time of DFAT cells was observed to be 65 h at passage 2, which decreases to 48 h at passage 10. DFAT cells demonstrate persistent proliferative potential even at passage 11, with a low frequency of cellular senescence. However, the proliferative capacity of DFAT cells isolated from donors over the age of 70 years old may be decreased. Despite this, successful expansion of DFAT cells has been reported for a wide range of donor ages, ranging from 4 to 81 years old [11].

Besides the standard “ceiling culture” system described previously, several groups developed modified approaches to study DFAT cells. Jumabay et al. described an alternative method for isolating DFAT cells that does not involve the use of ceiling culture (Fig. 2B). Adipocytes were harvested from the tissue and incubated in culture medium for 24 h, following which the cells were transferred to a new dish with a 70 mm insert filter. The DFAT cells derived from the adipocytes were allowed to sink through the filter and were collected after 5 days. The inclusion of these additional steps not only enhances the purity of DFAT cells but also significantly increases the early expression of pluripotency markers [33].

Prior to this, Fernyhough et al. had investigated several techniques, including differential plating (Fig. 2C, D), to improve the purity of DFAT cells. Differential plating is based on the differential attachment times of mature adipocytes and other cell types to the culture flask, thereby enabling the separation of contaminating cells. After 1–2 days of ceiling culture, mature adipocytes are suspended in the medium while non-lipid-containing cells attach to the bottom surface. In early differential plating (Fig. 2C), the mature adipocytes in the medium are transferred to a new flask, leaving the attached contaminating cells behind. Following 3–4 days of ceiling culture, mature adipocytes adhere to the top surface. In late differential plating (Fig. 2D), the cells attached to the ceiling are trypsinized and centrifuged after 3–4 days of ceiling culture to eliminate the contaminating cells that have been culturing along with the mature adipocytes due to the buoyant nature of adipocytes [32]. This protocol, by taking advantage of the increased time needed for adipocyte adherence compared to the contaminating SVF, allows the generation of a pure population of DFAT cells. The models developed by Jumabay [33] and Fernyhough [32] are likely to increase the homogeneity of DFAT cells. Despite the advancements in isolation techniques, obtaining a pure population of adipocytes for downstream DFAT cell production remains a challenge. Ensuring high purity of DFAT cells is essential for their clinical application in the future.

### Identification of DFAT cells

At present, the phenotype and specific surface markers for DFAT cells are controversial. Current studies suggested that DFAT cells are positive for CD13, CD29, CD44, CD49d, CD73, CD90, and CD105, and negative for CD11b,CD14, CD31, CD34, CD45, CD106, and HLA-DR [34] (Table 1). DFAT cells, ASCs, and other types of MSCs have similar surface markers, but there may be some differences in their expression levels. MSCs, including adipose-derived stem cells, express CD105, CD73, and CD90, but lack the expression of CD45, CD34, CD14, CD11b, CD79a, CD19, and HLA-DR. These markers are considered to be characteristic of MSCs and are used to identify them [34]. DFAT cells are a subtype of adipose-derived stem cells that have been dedifferentiated from mature adipocytes. DFAT cells have been found to express similar surface markers to adipose-derived stem cells, including CD105, CD73, and CD90, but may also express some markers that are not typically found in other MSCs, such as CD36 and CD10 [7]. In addition, DFAT cells have a higher expression of the hematopoietic progenitor marker CD34 and pericyte markers NG2 and α-SMA compared to ASCs, possibly due to their origin from the vascular stromal fraction of adipose tissue that is enriched in pericytes [35, 36].

**Table 1:** Surface markers for identification of DFAT cells

surface markers	Name	Category	Positive/negative
CD13	Aminopeptidase	Surface enzyme	Positive
CD73	Ecto-5’-nucleotidase	Surface enzyme	Positive
CD29	β_1_ integrin	Adhesion molecule	Positive
CD49d	α_4_ integrin	Adhesion molecule	Positive
CD105	Endoglin	Adhesion molecule	Positive
CD44	Hyaluronate	Receptor molecule	Positive
CD90	Thy-1	Extracellular matrix	Positive
CD11b	α_b_ integrin	Adhesion molecule	Negative
CD31	PECAM-1	Adhesion molecule	Negative
CD106	VCAM-1	Adhesion molecule	Negative
CD14	Lipopolysaccharide	Receptor molecule	Negative
CD45	Leukocyte common antigen	Receptor molecule	Negative
CD34	Hematopoietic progenitor cell antigen	Stem cell	Negative
HLA-DR	Human leukocyte antigen DR	Histocompatibility antigen	Negative

PECAM-1: platelet endothelial cell adhesion molecule-1; VCAM-1: vascular cell adhesion molecule-1

Overall, while there is some overlap in surface marker expression between DFAT cells, ASCs, and other types of MSCs, there are also differences that reflect their tissue-specific origin and potentially different functional properties.

DFAT cells have been reported to express several markers typically associated with ESCs, including Oct4, SOX2, c-Myc, and Nanog, which are known to play a critical role in maintaining pluripotency [37]. Moreover, DFAT cells also exhibit high alkaline phosphatase and telomerase activity, providing additional evidence of their similarities to undifferentiated pluripotent stem cells. However, it has been observed that the expression of pluripotency markers in DFAT cells decreases significantly after being cultured for more than two weeks. Previous studies that have focused on specific lineages may have overlooked the early expression of pluripotency markers in DFAT cells, potentially leading to the underestimation of their pluripotent potential [38–40]. Matsumoto et al. discovered that, similar to BMSCs and ASCs, DFAT cells express only HLA-ABC and not HLA-DR [11].

Thus, owing to their favorable safety profile and diminished immunogenicity, DFAT cells may present an appealing mesenchymal source for a range of cell-based therapies and tissue engineering applications.

## Function of DFAT cells

### Multilineage potential of DFAT cells

It is widely believed that DFAT cells could redifferentiate in cells belonging to this lineage (adipocytes, osteoblasts, chondrocytes) and therefore might be characterized as multipotent [41–43].In addition to the above differentiation directions, the study by Kazama et al. shows that the regulatory region of MYOD1, a master regulator for skeletal myogenesis, in DFAT cells is highly methylated [44]. However, treatment with a demethylating agent, 5-azacytidine, led to the expression of MYOD1 and MYOG in DFAT cells, as well as the formation of multinucleated cells expressing MUTYH, indicating muscle lineage differentiation [44]. Another study demonstrated that DFAT cells could differentiate into smooth muscle cells when cultured with DMEM containing 5% FCS and 5 ng/ml TGF (transforming growth factor)-β1. The expressions of TAGLN2, α-smooth muscle actin (α-SMA), and smooth muscle-myosin heavy chain were increased in DFAT cells during the first week of differentiation culture [45]. Furthermore, the article discusses how DFAT cells could differentiate into cardiomyocytes when cultured alongside neonatal cardiomyocytes or grown on semi-solid methylcellulose medium. DFAT cells were found to express specific markers indicative of cardiomyocyte differentiation, including GATA4 and Nkx2.5 nuclear proteins, cardiac sarcomeric actin, and troponin-T [46]. Taken together, the findings suggest that DFAT cells have the potential to differentiate into multiple cell lineages, indicating that DFAT cells are pluripotent [35, 47, 48].

### Secretory abilities of DFAT cells

Similar to other stem cells, DFAT cells have a powerful secretory function. Perrini et al. demonstrated that DFAT cells from obese individuals were able to secrete multiple cytokines and growth factors, including interleukin (IL)-1β, IL-1ra, IL-6, IL-8, IL-15, IL-17, granulocyte colony-stimulating factor (G-CSF), interferon (IFN)-γ, tumor necrosis factor (TNF)-α, eotaxin, monocyte chemoattractant protein-1 (MCP1), and vascular endothelial growth factor (VEGF) [49]. Poloni et al. obtained similar results using DFAT cells from nonobese individuals [50]. The secretome of DFAT cells would be altered under different conditions. Watanabe et al. found that DFAT cells could secrete large amounts of angiogenic factors such as VEGF and hepatocyte growth factor (HGF) when cultured under hypoxic conditions, and the secretion amount was significantly higher than that of ASCs and fibroblasts. When co-cultured with endothelial cells, DFAT cells further enhanced the expression of several angiogenic factors like HGF, TGF-β, and fibroblast growth factor 2 while the gene expression of mature pericyte-specific markers (α-SMA, NG2 and PDGFRβ) was largely increased [51]. The secretory profile of DFAT cells includes a wide range of cytokines[49], and under specific media conditions, DFAT cells secrete related proteins to promote recovery from lesion models, demonstrating the potential therapeutic effect of DFAT cells for regenerative medicine and tissue engineering.

## Regenerative medicine based on DFAT cells

Owing to their capacity for multilineage differentiation and secretory activities, DFAT cells hold great promise for therapeutic applications in a range of degenerative and injury-related disorders. Here, we summarized the recent research developments concerning the use of DFAT cells in diverse animal models of disease, as presented in Table 2. Table 2 covers the application indication, cell sources (adipocytes from rat, mouse, rabbit, and human), routes of administration (surgical implantation, local injection, intravenous injection, etc.), and therapeutic results. Despite the encouraging preclinical results, clinical trials of DFAT cells remain limited at present.

**Table 2:** Summary of in vivo application of DFAT cells in animal disease model

	Application	DFAT cells source	Administration route	Animal model	Grouping (EG and CG)	Results
Nakano et al. [58]	Bone	Rat	DFAT Cells + aPRP + GS (Surgical implantation)	Rat	EG: ①DFAT cells + scaffold	The DFAT cell proliferation rate was significantly increased by the addition of aPRP, with significantly higher Runx2 and OCN expression levels than those in the controls
					②DFAT cells + scaffold + aPRP	
					CG: no treatment	
Shirakata Y et al. [59]	Bone	Rat	DFAT Cells + PLGA/HA (Surgical implantation)	Rat	EG: DFAT cells + scaffold	Combined with PLGA/HA composite to promote bone regeneration
					CG: no treatment	
Kikuta et al. [60]	Bone	Rabbit	DFAT Cells + β-tricalcium phosphate/type I collagen sponge (Surgical implantation)	Rabbit	EG: DFAT cells	Promoted bone regeneration and alleviated ovariectomy-induced osteoporosis
					CG: Saline	
Yanagi et al. [61]	Bone	Rat	DFAT cells from 3D spheroids (Surgical implantation)	Rat	EG: 3D spheroid DFAT cell	The transplantation of DFAT cells from 3D spheroids accelerated bone healing
					CG: ①2D monolayer DFAT cell	
					②collagen sponge	
Nakayama et al. [64]	Cartilage	Rat	Cell Suspension (local injection)	Rat	EG: DFAT cells	DFAT cells differentiate into NP-like cells and induced ectopic formation of nucleus pulposus-like tissue
					CG: PBS	
Jumabay et al. [33]	Myocardium	Rat	Cell Suspension (local injection)	Rat	EG: DFAT cells	DFAT cells convert to cardiomyocyte phenotype and repair infarcted cardiac tissue
					CG: Saline	
Obinata et al. [68]	Skeletal muscle	Rat	Cell Suspension (local injection)	Rat	EG: DFAT cells	Recruited macrophages and transformed into SMC phenotype, leading to a significant increase in the thickness of the damaged urethral sphincter
					CG: Saline	
Sakuma et al. [45]	Smooth muscle	Human	Cell Suspension (local injection)	Mice	EG: DFAT cells	DFAT Cells can differentiate into Smooth muscle-like cells and contribute to bladder tissue regeneration
					CG:Hanks’ balanced solution	
Ikado et al. [69]	Ureteropelvic epithelial cells	Rat	Cell Suspension (local injection)	Rat	EG: DFAT cells	Inhibited VUR-induced tissue damage, such as ureteral dilatation and renal cell apoptosis
					CG: Saline	
Watanabe et al. And Planat-Benard et al. [36, 51]	Endothelium	Mouse	Cell Suspension (local injection)	Mice	EG: DFAT cells	DFAT cells improved blood flow in the ischemic hindlimbs more than ASCs
					CG: ①Saline	
					②ASCs	
Soejima et al. And Asami et al. [70, 71]	Endothelium	Rat	Cell Suspension (local injection)	Rat	EG: ①DFAT cells	Combined with bFGF to shorten the time required for angiogenesis and skin regeneration
					②DFAT cells + bFGF	
					CG: no treatment	
Kashimura et al. [72]	Endothelium	Rat	Cell Suspension (local injection)	Rat	EG: DFAT cells	The submuscular connective tissue of the skin was thickened with visible angiogenesis
					CG: no treatment	
Mikrogeorgiou et al. [75]	Nerve	Rat	Cell Suspension (Intravenous injection)	Rat	EG: DFAT cells	Relieved inflammation in acute phase of brain injury
					CG: Ringer’s bicarbonate solution	
Kakudo et al. [76]	Nerve	Human	Cell Suspension (Intravenous injection)	Mice	EG: DFAT cells	Restored part of cerebral cortical function
					CG: PBS	
Yamada et al. [77]	Nerve	Mouse	Cell Suspension (local injection)	Mice	EG: DFAT cells	Neuroremyelination and inhibited glial scar formation for the recovery of hindlimb motor function
					CG: DMEM	
Matsumine et al. And Fujimaki et al. [78, 80]	Nerve	Rat	DFAT Cells + nerve conduits (Surgical implantation)	Rat	EG: DFAT cells + conduits	Filled in artificial nerve conduits to promote axonal growth and enhance its maturity and physiological function
					CG: ①only conduits	
					②type I collagen + conduits	
Ishioka et al. [82]	Colon	Human	Cell Suspension (Intraperitoneal injection)	mice	EG: DFAT cells	Inhibited T cell-mediated cellular inflammation
					CG: PBS	
Maruyama et al. [84]	Kidney	Rat	Cell Suspension (Renal artery injection;	Rat	EG: DFAT cells	Reduced proteinuria and relieved glomerulosclerosis and interstitial fibrosis through inhibited TSG-6-mediated immune
			Tail vein injection)		CG: Saline	
Nur et al. [84]	Kidney	Mouse	Cell Suspension (Intravenous injection)	Mice	EG: DFAT cells	DFAT cells reduced the expression levels of TGF-β1 and fibronectin mRNAs
					CG: PBS	
Sugawara et al. And Akita et al. [87, 88]	Periodontal fenestration defects	Rat	DFAT Cells + atelocollagen or PLGA (Surgical implantation)	Rat	EG: DFAT cells + scaffold	DFAT cells were found to have greater potential for promoting periodontal tissue regeneration than ASCs
					CG: ①ASCs + scaffold	
					②only scaffold	

EG: experimental group; CG: control group; aPRP: activated platelet-rich plasma; GS: gelatin sponges; Runx2: Runt-related transcription factor 2; OCN: osteocalcin; PLGA/HA: polylactic acid-glycolic acid/hydroxyapatite; 3D: Three-dimensional; 2D: two-dimensional; PBS: phosphate-buffered saline; SMC: smooth muscle cell; YUR: vesicoureteral reflux; bFGF: basic fibroblast growth factor; DMEM: Dulbecco’s modified Eagle’s medium; TSG: TNF-stimulated gene; TGF: transforming growth factor

### Bone regeneration

The bone regeneration strategies based on DFAT cells have great potential as DFAT cells can differentiate into osteoblasts [52]. Compared with ASCs isolated from BFP, the DFAT cells had higher osteoblastic differentiation markers such as bone- specific alkaline phosphatase (ALP), osteocalcin, and calcium deposition [53]. Tsurumachi et al. found that the dedifferentiation efficiency of mature adipocytes was size-dependent. DFAT cells from small adipocytes exhibited higher osteogenic potential than those from large adipocytes [54]. Bone regeneration involves a complex interaction between DFAT cells and biological factors [55]. DFAT cells undergo osteogenic differentiation by the stimulated of retinoic acid, an analogue of retinol that interacts with bone morphogenetic proteins (BMPs) to limit adipogenesis and promote osteogenesis [43]. Fujisaki et al. found that in the process of osteogenesis, BMP-2 stimulated DFAT cells and ASCs in different pathways, respectively. BMP-2 activated the phosphorylation for the expression of ERK1/2 and Smad2 in DFAT cells during osteogenesis, instead of Smad1/5 in ASCs [56]. Additionally, epigallocatechin-3-gallate (EGCG) also affected the production of osteogenic factors [57]. To be ideal bone graft substitutes, scaffolds must be biodegradable and biocompatible and exhibit strong osteoinductive properties. Some studies reported that DFAT cells maintained well osteogenic differentiation ability in polylactic acid-glycolic acid/hydroxyapatite (PLGA/HA), β-tricalcium phosphate/type I collagen, and gelatin sponges [58–60]. Even without scaffolding, DFAT cells promote osteoblast differentiation and new bone formation by establishing three-dimensional (3D) spheroid [61].

### Cartilage repair

Repair of cartilage injury or intervertebral disc degenerative disease is a major problem owing to the weak self-healing ability of cartilage tissue [62]. DFAT cells with chondrogenic differentiation potential have attracted the attention of researchers. In vitro studies have showed that chondrogenic induction was facilitated by the addition of L-ascorbic acid-2-phosphate, proline, pyruvate, and TGF-β3 [60]. However, in the process of inducing DFAT cells to differentiate into cartilage, there is no effective induction method so far. Okita et al. found that adding an appropriate amount of strontium ions (Sr) to the chondrogenesis-inducing medium significantly promoted the early differentiation of DFAT cells into chondrocytes [63]. This may be a feasible method to enhances the differentiation of DFAT cells into cartilage for cartilage regeneration therapy. Nakayama et al. found that DFAT cells injected into the intervertebral disc could form a nucleus pulposus (NP)-like matrix in the outer layer of the annulus fibrosus. DFAT cells directly differentiated into NP-like cells and induce ectopic formation of NP-like tissue to enhance the regeneration of the intervertebral disc [64]. Currently, few studies tried to combine DFAT cells with scaffolds, which will be a potential direction of cartilage regeneration medicine in the future.

### Myocardium repair

Cardiovascular disease(CVD) is the leading cause of death globally and can lead to ischemia in critical regions [65]. DFAT cells expressed cardiac phenotype markers (Nkx2.5, troponin-T, GATA4, and cardiac actin) when co-cultured with cardiomyocytes and also when grown in MethoCult medium in the absence of cardiomyocytes. In a mouse model of acute myocardial infarction, intramyocardially injected DFAT cells accumulated and expressed myocardial sarcomeric proteins in the infarcted myocardium and form myotube-like structures. Concurrently, there was a significant increase in capillary density within the infarcted area. [46]. These results demonstrated that DFAT cells have the ability to differentiate to cardiomyocyte-like cells in vitro and in vivo, suggesting that DFAT cells represent a promising candidate cell source for cardiomyocyte regeneration in severe ischemic heart disease [46].

### Muscle regeneration

Stress urinary incontinence (SUI) is a common type of incontinence in women [66]. Rat models of vaginal distension (VD) have been utilized to replicate the symptoms of SUI, as this technique leads to the destruction and degeneration of the urethral sphincter, which is composed of both striated and smooth muscle [67]. DFAT cell transplantation has been shown to recruit macrophages to facilitate the thickening of the striated muscle layer, and to directly differentiate into smooth muscle cell (SMC) phenotypes to promote the thickening of the smooth muscle layer [68]. DFAT cells could differentiate into smooth muscle cells in cryo-injured bladder, which was helpful for the regeneration of the injured bladder tissue [43]. Ikado et al. found in a severe vesicoureteral reflux (VUR) model (the model did not cause damage to the smooth muscle tissue at the vesicoureteral junction) that transplanted DFAT cells effectively inhibited VUR-induced tissue damage, such as ureteral dilatation and renal cell apoptosis to exerted nephro protective effects [69].

### Blood vessel regeneration

The lack of blood perfusion ultimately limits tissue function. Watanabe et al. confirmed the possibility that DFAT cells could differentiate into pericyte-like cells via the TGF-β1-Smad2/3 signal transduction pathway and secretion of angiogenic factors when co-cultured with endothelial cells [51]. Blood flow to ischemic muscle tissue was significantly improved after DFAT cell transplantation. This was accompanied by an increase in the density of mature blood vessels that are positive for both IB4 and α-SMA, indicating that DFAT cells have the potential to augment neovascularization and promote vessel maturation [36, 51]. The capacity of DFAT cells to differentiate directly into SMC phenotype might also underlie the observed functional recovery of ischemic muscle tissue [68]. Soejim et al. and Asami et al. both demonstrated that mixing DFAT cells with basic fibroblast growth factor (bFGF) in artificial dermis (AD) transplantation/full-thickness skin grafts considerably shortened the time required for angiogenesis and skin regeneration [70, 71]. What’s more, DFAT cells can directly differentiate into endothelial cells, which greatly enhance the vascularization of the flap graft area and promotes flap survival [72].

### Nervous system regeneration

Cerebral ischemia is a common clinical disease in which partial or complete interruption of local blood flow in the brain and a series of pathological and biochemical events lead to neurological and motor dysfunction [73]. Studies showed that DFAT cells may have a better ability to differentiate into oligodendrocytes, astrocytes, and neuron-like cells in comparison with ASCs [74]. Mikrogeorgiou et al. found that, DFAT cells can cut down acute injury markers, and release of neurotrophic factors (IGF-1 and NGF) to reduce neuronal apoptosis. Intravenously injected DFAT cells restores a part of cerebral cortical function, but does not reduce the cerebral infarct area [75]. Moreover, labeled DFAT is mainly distributed in other organs, such as spleen, lung and liver, considering future transplantation methods such as intra-arterial or intracerebral administration rather than intravenous administration [75, 76]. Yamada et al. pointed out that DFAT cells may enhance neuroremyelination and inhibit glial scar formation in spinal cord injury (SCI) mice through cell-autonomous and non-autonomous effects while improving the limb movement ability after SCI [77].

Recent studies have reported that DFAT cells can differentiate into Schwanncells and facilitate native Schwann cell activity [78]. To bridge nerve defects, scientists have focused on nerve conduits combined with DFAT cells [79]. Matsumine et al. filled DFAT cells in silicone tubes and transplanted them into a rat model with a 7 mm defect in the buccal branch of the facial nerve. The nerve fiber diameter, axon diameter and myelin thickness in the DFAT cell transplantation group were significantly higher than those in the control group. Moreover, the immunocolocalization results indicated that the regenerated nerves in the DFAT cell group had Schwann-like supportive cells that were double positive for S100 and GFP [78]. Now polyglycolic acid (PGA) conduit can further promote the neurite regeneration ability of DFAT cells [80].

### Regulation of autoimmune diseases

Autoimmune diseases are a class of diseases in which the immune balance of the body is disrupted, causing damage to the body, including systemic lupus erythematosus (SLE), rheumatoid arthritis (RA), inflammatory bowel disease (IBD), etc. [81]. Various studies have demonstrated the potential role of DFAT cells in regulating inflammation. Ishioka et al. conducted co-culture experiments involving CD3 + T cells and DFAT cells, where they observed that DFAT cells could inhibit T cell proliferation in a density-dependent manner. The expression of genes responsible for immunosuppression, namely HGF, TRAIL, IDO1, Ptgs2, and NOS2, was significantly increased in DFAT cells when exposed to proinflammatory factors like IFNγ, IFNβ, or TNFα, particularly TNFα. These findings suggest that DFAT cells possess the potential to modulate the immune response and suppress T cell proliferation [82]. In the context of lupus nephritis, abnormal regulation of renal inflammation can lead to glomerular damage and reduced renal function [83]. To evaluate the therapeutic potential of DFAT cells in treating kidney injuries, a study was conducted where DFAT cells were transplanted into a kidney injury model. The results showed that transplantation of DFAT cells could reduce tissue inflammatory response and improve renal insufficiency by inhibiting the TNF-stimulated gene (TSG-6)-mediated immune response [84]. Furthermore, in another study, the positive therapeutic effect of DFAT cell transplantation was investigated in the context of renal dysfunction induced by habu snake venom (HSV). The findings indicated that transplantation of DFAT cells could alleviate the adverse effects of HSV-induced renal dysfunction [85].

### Application of dentistry

Periodontal defects are primarily caused by periodontal disease and trauma, resulting in the loss of cementum and alveolar bone tissue [86]. Recent studies have investigated the potential of utilizing DFAT cells to promote periodontal tissue regeneration. Specifically, when DFAT cells were transplanted into a periodontal tissue defect model in combination with atelocollagen scaffolds or PLGA, the transplanted DFAT cells demonstrated active proliferation and were able to promote the regeneration of alveolar bone, periodontal ligament, and cementum. As a result, the regenerated periodontium exhibited a similar architectural arrangement to the original tissue [87, 88]. Notably, DFAT cells were found to have greater potential for promoting periodontal tissue regeneration than ASCs [88].

## Conclusion

**Fig. 3:**
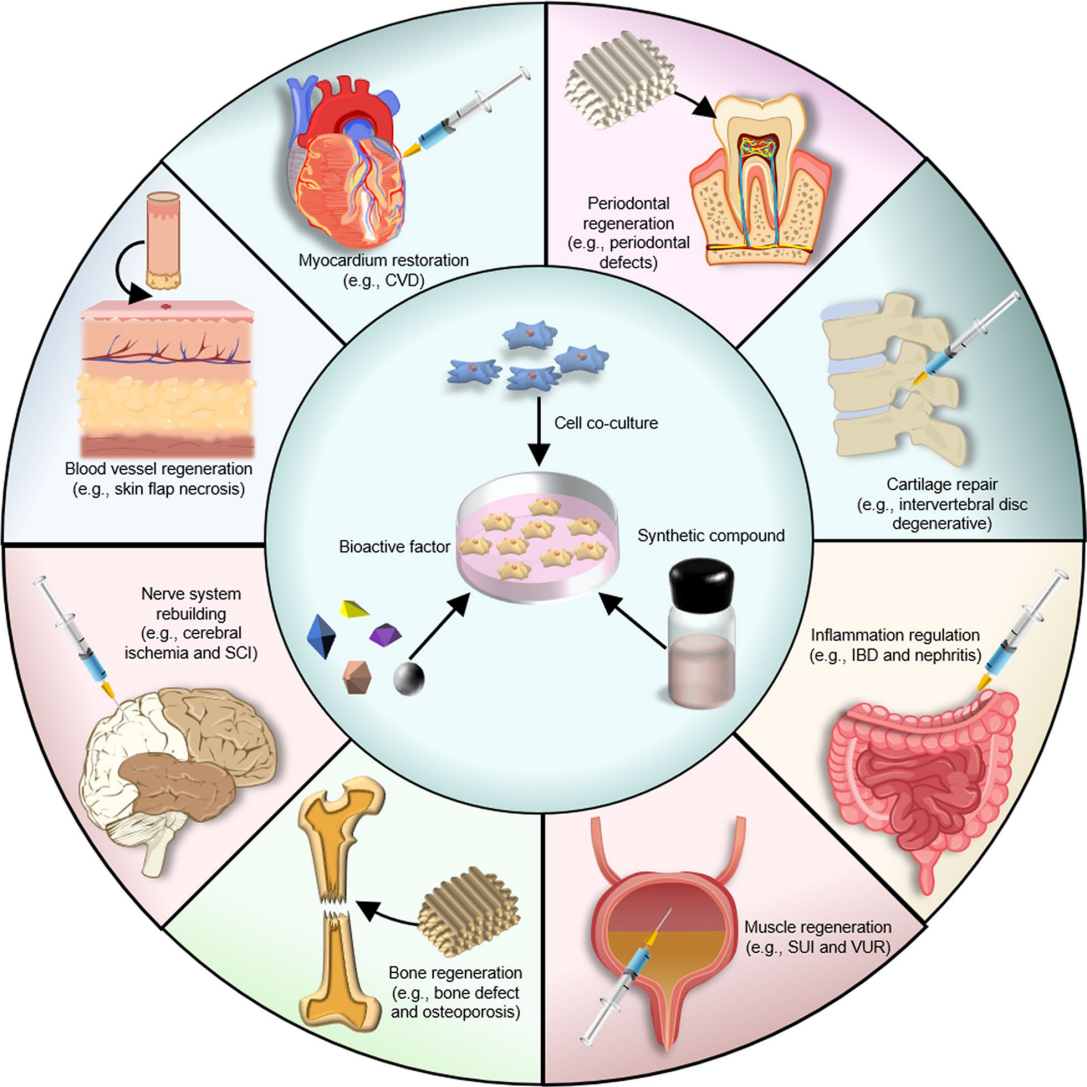
Schematic representation of the potential uses of DFAT cells in regenerative medicine. CVD: cardiovascular disease; IBD: inflammatory bowel disease; SUI: stress urinary incontinence; VUR: vesicoureteral reflux; SCI: spinal cord injury. In Fig. 3, all the images except for "the skin structure" sourced from https://biorender.com/ were self-described

DFAT cells are derived from mature adipocytes and can be conveniently obtained via standard or optimized “ceiling culturing” techniques. DFAT cells demonstrate a high degree of homogeneity and express typical stem cell surface markers such as CD13, CD29, CD44, CD49d, CD73, CD90, and CD105. With the ability of multilineage differentiation and potent secretory function, DFAT cells will be the promising candidates for tissue regeneration engineering and regenerative medicine, applied to the treatment of clinical problems such as cardiac and kidney diseases, autoimmune disease, soft and hard tissue defects (Fig. 3).

## Data Availability

Not applicable.

## Abbreviations

DFAT: Dedifferentiated fat
BMSCs: Bone marrow mesenchymal stem cells
ASCs: Adipose-derived stem cells
ESCs: Embryonic stem cells
iPSCs: Induced pluripotent stem cells
MAs: Mature adipocytes
SVF: Stromal vascular fraction
FBS: Fetal bovine serum
UC-MSCs: Umbilical cord-derived MSCs
TGF: Transforming growth factor
CD: Clusters of differentiation
α-SMA: α-Smooth muscle actin
IL: Interleukin
G-CSF: Granulocyte colony stimulating factor
FN: Interferon
TNF: Tumor necrosis factor
MCP1: Monocyte chemoattractant protein-1
VEGF: Vascular endothelial growth factor
HGF: Hepatocyte growth factor
BTE: Bone tissue engineering
BFP: Buccal fat pad
ALP: Alkaline phosphatase
BMP: Bone morphogenetic protein
aPRP: Activated platelet-rich plasma
PLGA/HA: Polylactic acid-glycolic acid/hydroxyapatite
TEB: Tissue engineered bone
2D/3D: Two or three-dimensional
IFP: Infrapatellar fat pad
SC: Subcutaneous adipose tissue
Sr: Strontium ions
NP: Nucleus pulposus
IDD: Intervertebral disc degeneration
CVD: Cardiovascular disease
MI: Myocardial infarction
LPP: Leak point pressure
VD: Vaginal dilation
SMC: Smooth muscle cell
VUR: Vesicoureteral reflux
GFP: Green fluorescence protein
IB4: Isolectin β4
PAD: Peripheral artery disease
ASO: Atherosclerosis obliterans
CLI: Limb ischemia
bFGF: Basic fibroblast growth factor
AD: Artificial dermis
IGF-1: Insulin-like growth factor
NGF: Nerve growth factor
SCI: Spinal cord injury
SLE: Systemic lupus erythematosus
RA: Rheumatoid arthritis
IBD: Inflammatory bowel disease
Th17: T helper cell 17
Treg: T regulatory cells
TSG: TNF-stimulated gene
HSV: Habu snake venom
PGA: Polyglycolic acid
